# Supplementary material for: netboxr: Automated discovery of biological process modules by network analysis in R
Source: PLoS One. 2020 Nov 2;15(11):e0234669. doi: 10.1371/journal.pone.0234669 (PMC7605689; doi:10.1371/journal.pone.0234669)

Functional annotation of modules by GO annotation enrichment using the enrichGO function of clusterProfiler (bioconductor.org/packages/release/bioc/html/clusterProfiler.html) **(A)** Without netboxr: GO annotation enrichment results with the entire input gene list (517 genes) **(B)** With netboxr: GO annotation enrichment on netboxr identified modules 1 to module 10. Modules 4, 8, and 9 do not have any significantly enriched (q<=0.05) GO annotations and therefore no figure is provided.

**(A)**

Using the entire input gene list


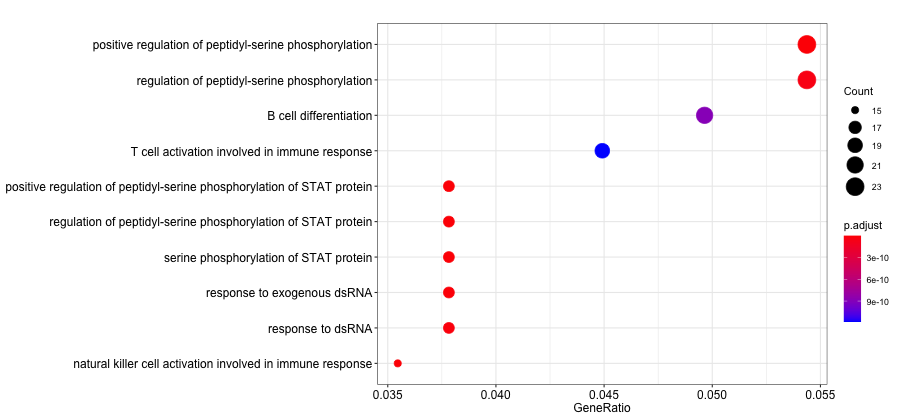


**(B)**

Module 1


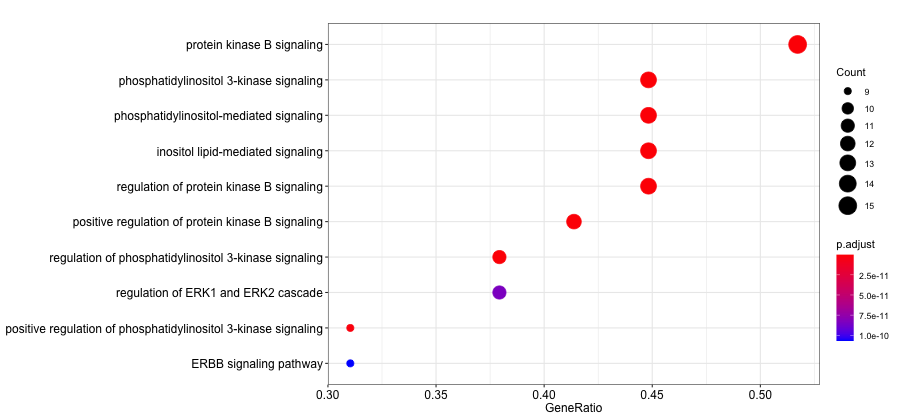


Module 2


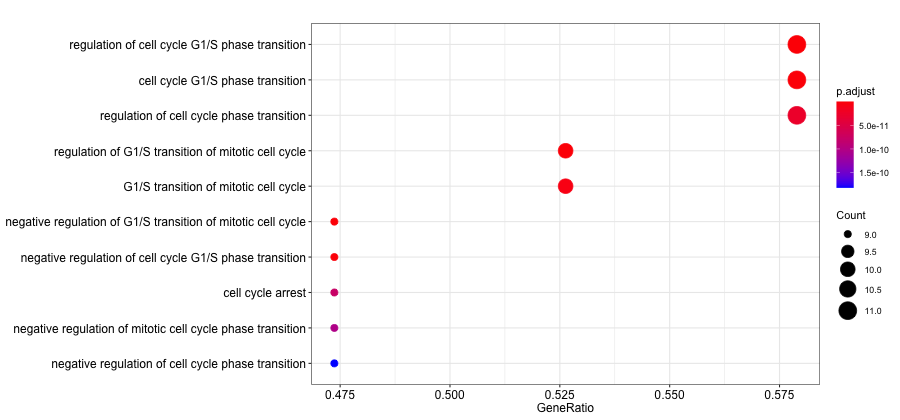


Module 3


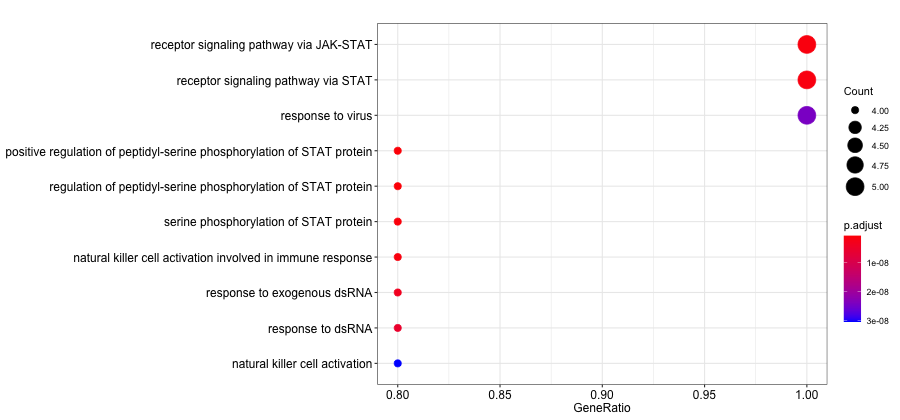


Module 5


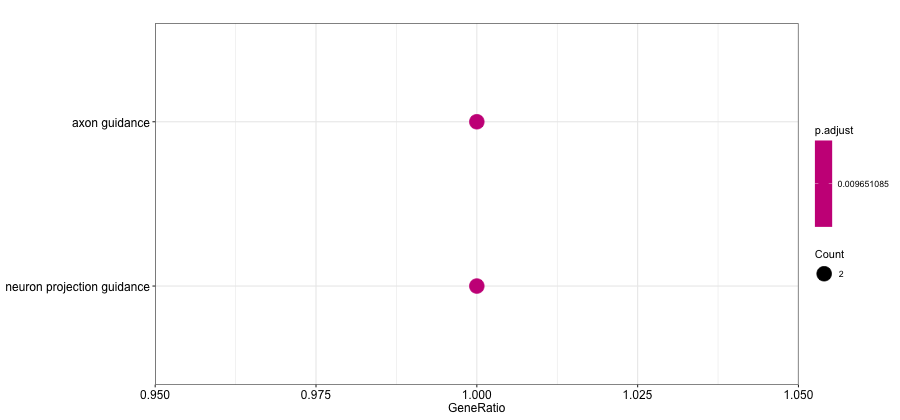


Module 6


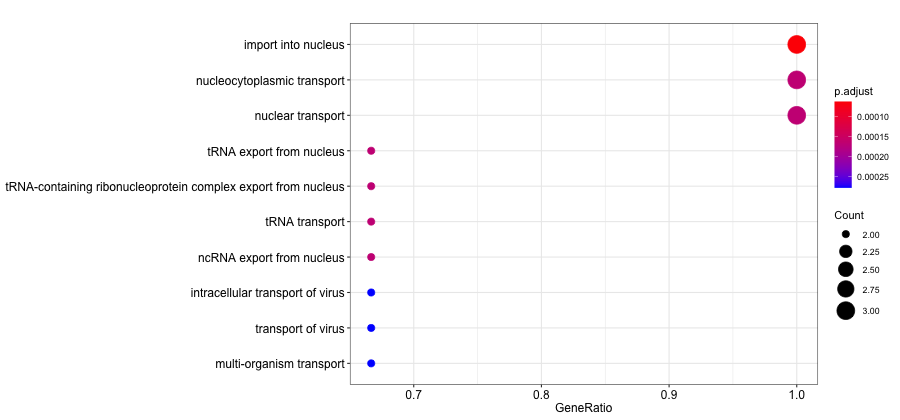


Module 7


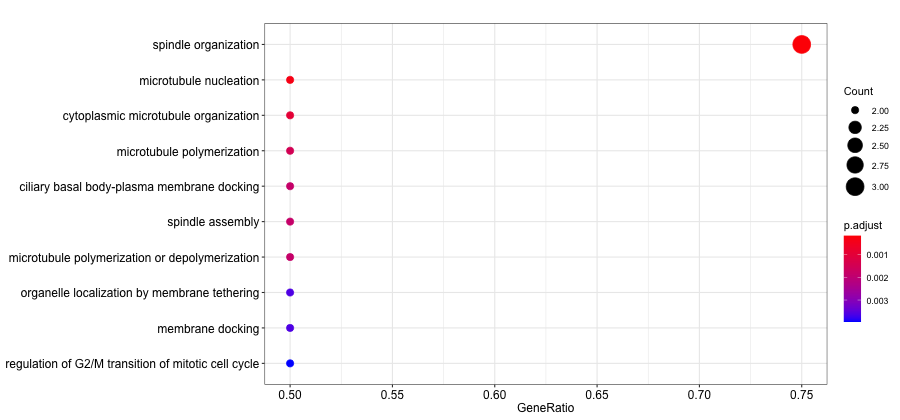


Module 10


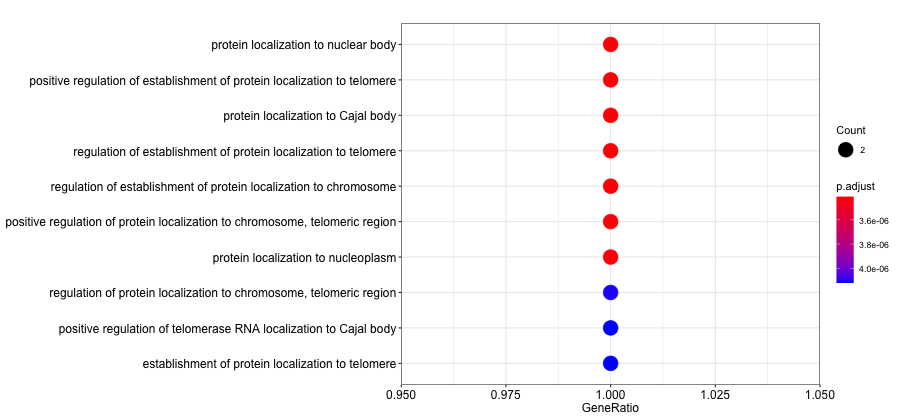

Supplement: S1 File — (DOCX) [file pone.0234669.s001.docx]
