# Supplementary material for: netboxr: Automated discovery of biological process modules by network analysis in R
Source: PLoS One. 2020 Nov 2;15(11):e0234669. doi: 10.1371/journal.pone.0234669 (PMC7605689; doi:10.1371/journal.pone.0234669)
Supplement: Attachment — Submitted filename: PONE-D-20-16149_comments.docx [file pone.0234669.s002.docx]

The authors developed an R package named NetBoxR that makes use of the NetBox algorithm to identify candidate cancer-related processes. The algorithm makes use of a network kbased approach that combines prior knowledge with a network clustering algorithm. This program can combine multiple data types, such as mutations and copy number alterations, leading to more reliable identification of functional modules. This tool can be useful for the research community to identify the functional modules in cancer data analysis.

I have some little concerns:

1. In the paper, it says” NetBoxR takes significantly altered genes from SNV mutation, copy number alteration associated with gene expression change, and merges them into a gene list for identification of pathway modules.” How can users to define the “significantly altered genes from SNV mutation, copy number alteration”, just like finding differentially expressed genes from expression profiles, how to take the variants into account to find these genes? Authors may provide some guidance on choosing these genes for a better performance in NetBoxR.

2. From the methodology side, there are differences between the GSEA algorithm and NetBox, it seems the NetBox overcomes some shortcomes, I suggest authors to do a comparison between the results from GSEA and NetBox, to provide the new findings from NetBox, which can not be got from GSEA.

3. In the online tutorial, the results from NetBox were used for GO enrichment, why not to do the GO enrichment at the first using the original gene list, what are the advantages by using the NetBoxR to do a pre-process before the GO enrichment?

4. In the result figure (paper, online), it shows some modules, but there is no annotation of these module? The author should show the function annotation for the modules in the result figures found by their program.

5. Minor: the text in original figure can be see clearly, but I cannot see any word in the figure embedded in the pdf file.
