# Supplementary material for: netboxr: Automated discovery of biological process modules by network analysis in R
Source: PLoS One. 2020 Nov 2;15(11):e0234669. doi: 10.1371/journal.pone.0234669 (PMC7605689; doi:10.1371/journal.pone.0234669)
Supplement: Attachment — Submitted filename: Response to reviewers.docx [file pone.0234669.s003.docx]

We thank the Editor and the reviewers for taking the time to review our paper and for the thoughtful comments and suggestions. We agree with almost all of them and have addressed them in this revision of the manuscript and in updates to netboxr on Bioconductor. Point by point responses are given below.

Reviewer 1(R.1)

**- R.1: General comment -**

**Reviewer Comment:** The authors developed an R package named NetBoxR that makes use of the NetBox algorithm to identify candidate cancer-related processes. The algorithm makes use of a network based approach that combines prior knowledge with a network clustering algorithm. This program can combine multiple data types, such as mutations and copy number alterations, leading to more reliable identification of functional modules. This tool can be useful for the research community to identify the functional modules in cancer data analysis.

**- R.1: Comment 1 -**

**Reviewer Comment:** In the paper, it says” NetBoxR takes significantly altered genes from SNV mutation, copy number alteration associated with gene expression change, and merges them into a gene list for identification of pathway modules.” How can users define the “significantly altered genes from SNV mutation, copy number alteration”, just like finding differentially expressed genes from expression profiles, how to take the variants into account to find these genes? Authors may provide some guidance on choosing these genes for a better performance in NetBoxR.

**Author Response:** Currently, in the example we provide, netboxr takes genes with significantly altered SNV mutations predicted by the “MutSigCV2” algorithm and genes with significantly altered copy numbers from “GISTIC2” into a gene list as input. While it is out of scope to provide an explanation for generating input gene lists under any circumstance, we now have two sets of instructions in our vignette (tutorial) for generating gene lists: 1) downloading gene lists for datasets in cBioPortal (which has ~200 cancer studies) and 2) a basic algorithm for generating an input gene list while pointing them to a corresponding TCGA publication with further details. We refer to this within the manuscript.

**Vignette link:**

Here is a direct link to the file: <https://www.bioconductor.org/packages/release/bioc/vignettes/netboxr/inst/doc/netboxrTutorial.html>

**Excerpt From Revised Manuscript:**

[Page 7] Additionally, within the vignette, we provide instructions for users to generate input data and retrieve input gene lists from publicly available studies on cBioPortal

**- R.1: Comment 2 -**

**Reviewer Comment:** From the methodology side, there are differences between the GSEA algorithm and NetBox, it seems the NetBox overcomes some shortcomings, I suggest authors do a comparison between the results from GSEA and NetBox, to provide the new findings from NetBox, which can not be got from GSEA.

**Author Response:** We address the comment in two ways: 1) we now include a figure (Figure 1) that gives a conceptual understanding of the differences between the use of algorithms such as NetBox and GSEA (and related: hypergeometric test), and 2) we include results that compare the use of the NetBox versus the hypergeometric test (Supplementary Information). Note: we use the hypergeometric test rather than GSEA; GSEA requires a ranked gene list whereas NetBox does not require this ranking information.

**Excerpt From Revised Manuscript:**

Unlike GSEA, NetBox is not limited to nor influenced by curated gene lists, it takes into account known interactions, and overcomes the issue of the occurrence of genes in more than one of the curated gene sets (Figure 1). The NetBox algorithm derives network modules de novo, based on the alteration data in tumor samples, such that the identified modules can not only recover or overlap with known pre-defined pathways but, more importantly, identify new functional gene groups that cross curated gene boundaries. For newly discovered modules, the functional annotation can then be derived from the annotation of the gene members, facilitating discoveries.

**- R.1: Comment 3 -**

**Reviewer Comment:** In the online tutorial, the results from NetBox were used for GO enrichment, why not to do the GO enrichment at the first using the original gene list, what are the advantages by using the NetBoxR to do a pre-process before the GO enrichment?

**Author Response:** Commonly used enrichment analyses performed via either a hypergeometric test or GSEA lose valuable information based on the topological relationships between input genes.

Pre-processing an enrichment analysis with the NetBox algorithm complements the more common work pipeline. In Supplementary Information, we now add enrichment results for the overall input gene list while also for the individual modules (Supplementary Information). Reviewers will now note that the overall enrichment results largely reflect the enrichment results of Module 3, with terms like “positive regulation of peptidyl−serine phosphorylation of STAT protein”, but that other modules also have coherent enrichments (e.g., regulation of cell cycle G1/S phase transition in Module 2) that could contribute to researchers’ understanding of their results.

Secondly, in our experience users are typically interested in the examination of molecular interactions between altered genes as a secondary analysis step after enrichment analyses to understand results in context; this is simplified by starting with network data provided by Pathway Commons, STRING, etc. that provide details for interactions of interest.

**Excerpt From Revised Manuscript:**

[Page 8] This module-driven exploration of the identified network allows a finer-grain understanding of the input gene list than through simply performing an enrichment analysis over the entire input gene list through 1) the topology of the identified network connections and 2) the annotation of specific modules (Figure 2, Supplementary Information).

**- R.1: Comment 4 -**

**Reviewer Comment:** In the result figure (paper, online), it shows some modules, but there is no annotation of these modules? The author should show the function annotation for the modules in the result figures found by their program.

**Author Response:** As part of Figure 2 we now include a descriptive process label for each module with accompanying information in Supplementary Information for how the label was determined.

**- R.1: Comment 5 -**

**Reviewer Comment:** the text in the original figure can be seen clearly, but I cannot see any word in the figure embedded in the pdf file.

**Author Response:** We have provided a high-resolution version of Figure 2 in the revised manuscript. Compiled PDFs usually have blurry figures embedded, but normally a link is provided to the high-resolution figure.

------------------------------------------------------------------------------

Reviewer 2 (R.2)

**- R.2: General comment -**

**Reviewer Comment:** Liu et al present the R package NetBoxR that implements the NetBox algorithm previously described in an article by Cerami et al (2010). This article presents little in terms of new methods, data or results; the main message is the implementation of the NetBox algorithm in R rather than a standalone command-line application.

The article is mostly written well and is easy to understand, at least if the reader is willing to refer to the Cerami et al work for some of the details.

**- R.2: Comment 1 -**

**Reviewer Comment:** NetBox and NetBoxR evaluate the significance of the found network by focusing on the largest module only. It would be beneficial to implement a method that can determine the significance of each module, not just the largest one.

**Author Response:** We thank Reviewer 2’s comment. There was a misunderstanding due to the incorrect use of graph terminology in the manuscript.

We have corrected the description of the statistical significance assessment section in the manuscript and in the vignette to replace the phrase “largest module” with the “largest connected component”.

To assess the global statistical significance of the identified network (the “identified network” is the entirety of the network shown in Figure 2), the NetBox algorithm tests the significance of the identified network based on the level of “global connectivity”. We have corrected the phrase “largest module” in the manuscript and the vignette (i.e., tutorial) to state the “largest connected component”.

Here, the “largest connected component” is composed of multiple interconnected modules M1, M2, M3, and M8 (Figure 2). Therefore, we do not access the significance of each identified module. Instead, this tests the overall statistical significance of the identified network. We apologize for the confusion in the previous manuscript and vignette.

See Reviewer 1 Comment 1 for a link to the vignette.

**Excerpt From Revised Manuscript:**

[Page 5] To assess the level of global connectivity, an empirical p-value is calculated by determining the number of times the size of the largest connected component (the largest network component can be composed of multiple modules) identified from the same number of randomly selected genes equals or exceeds the size of the largest connected component from the list of altered genes in the data set.

**- R.2: Comment 2 -**

**Reviewer Comment:** The identified modules are based on literature networks, which presumably makes any enrichment analysis in literature sets biased. If I understood the methods correctly, this inflation is the result of not just the connections that come from the literature, but also because linker genes are derived from the literature and included in the modules. It does not appear that the package contains any functions that could quantify and correct the bias.

**Author Response:** The reviewer is correct; there is no netboxr code to quantify or address literature bias in input networks. From our understanding of the field, there is no widely accepted method for correcting such a bias ("widely" here is defined by hundreds and thousands of citations; GSEA, for example, has 22000 citations and therefore to be considered "widely accepted"). Addressing this bias is outside of our project's work and in the absence of a widely accepted method, in the Conclusion, we mention the possibility of such biases and point to an appropriate reference that describes this and other biases that may confound the interpretation of any enrichment analysis.

**Excerpt From Revised Manuscript:**

[Page 8] As with any method, users should carefully consider the problems that they attempt to address and understand the strengths and limitations before using a particular method. Reimand et al. present a broader discussion of the biases that can be present in enrichment analyses, including the biases that can be present in existing pathway databases

**Implementation issues:**

**- R.2: implementation comment 1 -**

**Reviewer Comment:** As for implementation, a cursory reading of the help files indicates that they need to be improved. Reading the help for geneConnector, one learns for example that the argument networkGraph is “an igraph graph object” but it’s unclear what it is supposed to represent. Similarly, the argument communityMethod is described as “A string for community detection method c('ebc','lec')”. It is not clear what the shorthands mean. Arguments ‘directed’ and ‘keepIsolatedNodes’, although both logical, are described as “TRUE of FALSE” and “logic value”, respectively; neither has any information on what the TRUE and FALSE values actually determine. Similar comments apply to the description of the output of the function. The individual input arguments and output components need a description of what they represent, not just what type of R object is expected.

**Author Response:** We appreciate the reviewer's comment on the documentation for the geneConnector function in the package. We have revised the documentation of functions with similar documentation issues in the netboxr package for clarity.

**Documentation link:**

Here is a direct link to the file: <https://bioconductor.org/packages/release/bioc/manuals/netboxr/man/netboxr.pdf>

**- R.2: implementation comment 2 -**

**Reviewer Comment:** The authors say that the package provides “instructions for retrieving and processing genomic alterations such as SNV mutations and copy number alterations from data repositories such as cBioPortal (Cerami et al., 2012; Gao et al., 2013) or Genomic Data Commons (GDC) (Jensen et al., 2017)”. I did not find the instructions; perhaps I missed something in the vignette or in the help files?

**Author Response:** This was an oversight. See Reviewer 1 Comment 1 for a link to the vignette.

**Minor issues:**

**- R.2: Minor comment 1 -**

**Reviewer Comment:** The authors refer to the package as NetBoxR, but the package name on bioconductor is netboxr (all lowercase). This can cause confusion, e.g., NetworkManager::install(“NetBoxR”) produces an error.

**Author Response:** We have revised the package name in the manuscript to lowercase, “netboxr” to be consistent with the name in the Bioconductor repository.

**Excerpt From Revised Manuscript:**

[Page 1] netboxr: Automated Discovery of Biological Process Modules by Network Analysis in R

**- R.2: Minor comment 2 -**

**Reviewer Comment:** The sentence “ability to retrieve pathway data via the paxtoolsr package as available in ~20 data source aggregation Pathway Commons, (Luna et al., 2016) or STRING (Snel et al., 2000)” does not make sense.

**Author Response:** We have revised the sentence to clarify the pathway data retrieval in the netboxr package. The pathway can be retrieved either via paxtoolsr (for Pathway Commons) or other pathway data sources such as the STRING database as the input network for the netboxr package. This is discussed in the section “Alternative Pathway Data” of the vignette.

See Reviewer 1 Comment 1 for link to vignette.

**Excerpt From Revised Manuscript:**

[Page 6] 2) instructions to use pathway data via the paxtoolsr Pathway Commons package (Luna et al., 2016) or resources such as the STRING pathway database (Snel et al., 2000),

**- R.2: Minor comment 3 -**

**Reviewer Comment:** Contrary to what the authors seem to imply, the package does not seem to be available for R-3.6.3; one apparently needs R-4.0.0 or higher.

**Author Response:** We have revised the package requirement to R-4.0.0 or higher and removed reference to a particular R version in the manuscript (Bioconductor releases set the R version dependency and this can change with each release which happens twice a year).

**Excerpt From Revised Manuscript:**

[Page 7] netboxr was implemented in R and can be installed through the BiocManager package manager from Bioconductor (bioconductor.org).
