# Supplementary material for: netboxr: Automated discovery of biological process modules by network analysis in R
Source: PLoS One. 2020 Nov 2;15(11):e0234669. doi: 10.1371/journal.pone.0234669 (PMC7605689; doi:10.1371/journal.pone.0234669)
Supplement: Attachment — Submitted filename: PONE-D-20-16149_R1_comments.docx [file pone.0234669.s004.docx]

I did not find the response to the comments file in the review system (I am not sure if the authors forgot to submit it or something wrong with the review system).

I have read through the paper, and I can feel that authors has made great upgrade to their netboxr package, and refined the manuscript. It can be accepted.

One minor comment: There are too much kay words.
